# Magnetic resonance imaging findings in patients with polymyalgia rheumatica

**DOI:** 10.1590/0100-3984.2021.0151

**Published:** 2022

**Authors:** Renata Vidal Leão, Ana Luisa Garcia Calich, Isidio Calich, Marcelo Bordalo Rodrigues, Paulo Victor Partezani Helito, Denise Tokechi Amaral, Renata Fernandes Batista Pereira, Marcos Felippe de Paula Correa

**Affiliations:** 1 Hospital Sírio-Libanês, São Paulo, SP, Brazil.; 2 Instituto de Radiologia do Hospital das Clínicas da Faculdade de Medicina da Universidade de São Paulo (InRad/HC-FMUSP), São Paulo, SP, Brazil.

**Keywords:** Polymyalgia rheumatica/diagnostic imaging, Shoulder joint/pathology, Hip joint/pathology, Magnetic resonance imaging/ methods, Polimialgia reumática/diagnóstico por imagem, Articulação do ombro/patologia, Articulação do quadril/patologia, Ressonância magnética/métodos

## Abstract

**Objective:**

To describe the prevalence of magnetic resonance imaging (MRI) findings in patients with the clinical diagnosis of polymyalgia rheumatica (PMR).

**Materials and Methods:**

Sixteen consecutive patients with untreated PMR, meeting the American College of Rheumatology criteria, underwent MRI examinations of the shoulder(s), hip(s), or both, depending on clinical complaints. Six patients also underwent MRI of the spine.

**Results:**

We evaluated 24 shoulders, among which we identified subacromial-subdeltoid bursitis in 21 (87.5%), glenohumeral joint effusion in 17 (70.8%), and fluid distention of the long head of the biceps tendon sheath in 15 (62.5%). Peritendinitis and capsular edema were observed in 21 (87.5%) and 17 (70.8%) shoulders, respectively. We also evaluated 17 hips, identifying hip joint effusion in 12 (70.6%), trochanteric bursitis in 11 (64.7%), peritendinitis in 17 (100%), and capsular edema in 14 (82.4%). All six of the patients who underwent MRI of the spine were found to have interspinous bursitis.

**Conclusion:**

Subacromial-subdeltoid bursitis, glenohumeral joint effusion, and hip joint effusion are common findings in patients with PMR. In addition, such patients appear to be highly susceptible to peritendinitis and capsular edema. There is a need for case-control studies to validate our data and to determine the real impact that these findings have on the diagnosis of PMR by MRI.

## Abstract (pt)

**Objetivo:**

Descrever os achados de ressonância magnética (RM) mais prevalentes em pacientes com diagnóstico clínico de polimialgia reumática (PMR).

**Materiais e Métodos:**

Dezesseis pacientes com PMR não tratada, classificados pelos critérios do American College of Rheumatology, foram submetidos a RM do ombro e/ou quadril, segundo suas queixas clínicas. Seis pacientes também foram submetidos a RM da coluna.

**Resultados:**

Foram avaliados 24 ombros, identificando-se bursite subacromial-subdeltoide em 21 (87,5%), sinovite glenoumeral em 17 (70,8%) e distensão líquida da bainha do tendão da cabeça longa do bíceps em 15 (62,5%). Peritendinite e edema capsular foram observados em 21 (87,5%) e 17 (70,8%) ombros, respectivamente. Dezessete quadris foram analisados, identificando-se sinovite em 12 (70,6%), bursite trocantérica em 11 (64,7%), peritendinite em 17 (100%) e edema capsular em 14 (82,4%). Os seis pacientes que realizaram RM da coluna apresentavam bursite interespinhosa.

**Conclusão:**

Bursite subacromial-subdeltoide, sinovite glenoumeral e do quadril são achados de imagem prevalentes em pacientes com PMR. Além disso, achados como peritendinite e edema capsular tiveram alta prevalência nesses pacientes. Estudos de casocontrole devem ser realizados para validar esses dados e estabelecer o real impacto desses achados no diagnóstico de PMR.

## INTRODUCTION

Polymyalgia rheumatica (PMR) is a chronic inflammatory rheumatic disease that commonly affects women over 50 years of age^(1)^, and the incidence of the disease increases with advancing age^(2)^. In a large-scale study conducted in a predominantly white population in the United

States^(3)^, PMR was found to be the second most common inflammatory rheumatic disease, after rheumatoid arthritis (RA). One review of the literature, conducted in 2015, showed that the estimated prevalence rate for PMR in the United States was as high as 739 per 100,000, which suggests that there were 711,000 Americans with PMR^(2)^. The lifetime risk of developing the disease has been estimated to be 2.43% for women and 1.66% for men^(4)^.

The clinical presentation of PMR is pain and stiffness affecting the neck, shoulders, hips, and thighs. Constitutional symptoms, such as low-grade fever, weight loss, anorexia, and depression, occur in more than half of all patients with the disease. The onset of PMR is usually abrupt, and nocturnal pain is common among the affected individuals. The most sites of pain are the shoulders (in 70–95% of cases) and the pelvic girdle (in 50–70%), although the cervical and lumbar regions of the spine may also be affected^(5,6,7)^.

The current classification criteria for PMR are based on clinical and ultrasound findings. They include being over 50 years of age, presenting with bilateral shoulder pain, having an elevated C-reactive protein (CRP) level, and having an elevated erythrocyte sedimentation rate (ESR). The American College of Rheumatology/European League Against Rheumatism included ultrasound in the classification criteria for PMR^(1)^, which improved the specificity to discriminate between patients with and without the disease (Table 1). There is no specific confirmatory diagnostic test for PMR. Although markers of inflammation, such as elevated CRP level and ESR, are common findings in PMR, they are nonspecific^(8)^.

**Table 1:** Diagnostic criteria for PMR, established by the American College of Rheumatology/European League Against Rheumatism*, and the scoring algorithm.

Criterion	Score^†^	
	Without ultrasound (maximum of 6)	With ultrasound (maximum of 6)
Morning stiffness for > 45 min	2	2
Hip pain or limited range of motion	1	1
Absence of rheumatoid factor or anticitrullinated protein antibody	2	2
Absence of other joint involvement	1	1
At least one shoulder with subdeltoid bursitis, with or without biceps tenosynovitis and synovitis (either posterior or axillary), and at least one hip with synovitis, trochanteric bursitis, or both	Not applicable	1
Subdeltoid bursitis, biceps tenosynovitis, or synovitis, in both shoulders	Not applicable	1

* Prerequisite criteria: being over 50 years of age, having bilateral shoulder pain, having an elevated CRP level, and having an elevated ESR.

^†^ Without ultrasound, a score of 4 or more is categorized as PMR, whereas a score of 5 or more is categorized as PMR with ultrasound.

Source: Dasgupta et al.^(1)^.

The classical imaging findings in PMR include bilateral subacromial-subdeltoid (SASD) bursitis, trochanteric bursitis, and biceps tenosynovitis, as well as shoulder and hip synovitis. Those features are highly nonspecific and, in most cases, are not diagnostic, because they are frequently seen in older patients with degenerative or mechanical disorders. Periarticular soft-tissue edema has recently been described in patients with PMR and may add some specificity to the diagnosis^(7,9,10,11)^. Mori et al.^(7)^ suggested that the periarticular changes are the cause of the severe discomfort and myalgia in patients with PMR.

Because proximal pain and stiffness syndrome, a commonly accepted phenotype of PMR, can occur in many other rheumatic illnesses, as well as in patients with degenerative or mechanical musculoskeletal pain, the differential diagnosis of this clinical profile is broad. The main differential diagnoses are late-onset spondyloarthritis and RA^(12)^.

Despite its prevalence and clinical importance, the imaging patterns of PMR are not well known to radiologists, because there are few data regarding this disease in the radiology literature. Therefore, magnetic resonance imaging (MRI) scans of patients with PMR are often nondiagnostic. With the increasing number of MRI examinations performed in musculoskeletal radiology, radiologists will more frequently encounter imaging features suggestive of PMR and should be ready to recognize them. This study aims to determine the prevalence of MRI findings in patients with a clinical diagnosis of PMR.

## MATERIALS AND METHODS

### Study population

A total of 16 consecutive patients with untreated PMR, meeting the American College of Rheumatology criteria^(1)^, were identified by rheumatologists over a 30-month period (from August 2019 to December 2021). The inclusion criteria were being over 50 years of age; having bilateral shoulder pain; having an elevated CRP level, with or without an elevated ESR; and not having started anti-inflammatory treatment for PMR. Patients who had been diagnosed with another rheumatic disorder were excluded, as were those who were being treated with any anti-inflammatory agent.

All of the patients underwent MRI examinations of the shoulder(s), hip(s), or both, depending on the clinical complaints. Of the 16 patients evaluated, 14 underwent MRI of the shoulder (bilaterally in 10) and 10 underwent MRI of the hip (bilaterally in 7). Therefore, the final sample comprised 24 shoulders and 17 hips. Six patients also underwent MRI of the spine, which targeted the lumbar spine in four and the cervical spine in two.

Patient ages ranged from 50 to 81 years (mean, 67 years). Of the 16 patients in the sample, 11 (68.8%) were female and five (31.2%) were male. All of the patients had elevated ESRs and elevated CRP levels (ranges, 51–82 mm and 1.2–11.0 mg/dL, respectively).

### MRI acquisition parameters

The MRI examinations were performed in a variety of 1.5-T scanners—Aera (Siemens Healthineers, Erlangen, Germany); Espree (Siemens Healthineers); Avanto (Siemens Healthineers); Optima 450W (GE Healthcare, Milwaukee, WI, USA); 3.0-T scanners Achieva (Philips Medical Systems, Best, The Netherlands); Skyra (Siemens Healthineers); and HDX (GE Healthcare). All protocols were implemented as described in Tables 2 and 3. Intravenous contrast was not indicated for any of the patients, and contrast-enhanced images were acquired in only one shoulder and two hips (in three individuals for whom contrast was indicated for other examinations that were performed simultaneously).

**Table 2:** Image acquisition protocols for MRI of the shoulder.

Parameter	Plane/sequence				
	Coronal		Sagittal		Axial
	Proton density	T2wFS	T1-weighted	T2wFS	T2wFS
FOV (mm)	150–160	150–160	150–160	150–160	150–160
TR (ms)	2150–2900	2200–3000	340–740	2900–5900	2900–4300
TE (ms)	30–40	40–50	9–12	40–50	38–45
Slice thickness (mm)	3.0–3.5	3.0–3.5	3.0–3.5	3.0–3.5	3.0–3.5
Interslice gap (cm)	0.3–0.4	0.3–0.4	0.3–0.4	0.3–0.4	0.3–3.5

FOV, field of view; TR, repetition time; TE, echo time.

**Table 3:** Image acquisition protocols for MRI of the hip.

Parameter	Plane/sequence					
Coronal		Sagittal	Axial		Oblique
T1-weighted	T2wFS	T2wFS	T1-weighted	T2wFS	T2wFS
FOV (mm)	220	240	200	220–240	220–240	200
TR (ms)	510–624	2.2	2340–3290	489–705	2490–3030	2190
TE (ms)	13	38	38–44	13	38–44	35
Slice thickness (mm)	4.0	4.0	3.0–3.5	4.0–4.5	4.0–4.5	3.5
Interslice gap (cm)	0.3–0.4	0.3–0.4	0.3–0.4	0.3–0.4	0.3–0.4	0.3–3.5

FOV, field of view; TR, repetition time; TE, echo time.

### Imaging evaluation

The MRI scans were reviewed by a certified radiologist, with 15 years of experience in musculoskeletal radiology, who was blinded to the clinical data and diagnosis of the patients. The following imaging features were evaluated: SASD bursitis; glenohumeral joint effusion; fluid distention of the long head of the biceps (LHB) tendon sheath; hip joint effusion; trochanteric bursitis; periarticular soft-tissue edema (stratified into peritendinitis and capsular edema); and interspinous bursitis. The findings were qualitatively classified as present or absent.

The diagnosis of bursitis was based on fluid distention of the bursa, as identified on coronal T2-weighted fat-saturated (T2wFS) images of the shoulder and axial T2wFS images of the hip. In shoulders and hips, joint effusion was diagnosed by identifying abnormal fluid accumulation distending the joint capsule on coronal T2wFS images. Fluid distention of the LHB tendon sheath was also identified on axial T2wFS images of the shoulder; the diagnostic criterion was such distention being seen in at least two consecutive slices. Peritendinitis and capsular edema were diagnosed by detecting high signal intensity, primarily surrounding the tendons and in or around the capsule, respectively, on T2wFS images. Cervical and lumbar spine bursae were evaluated in order to identify any fluid collections.

### Statistical methods

The demographic and clinical characteristics of the study participants are expressed as means and ranges. The prevalence of imaging findings was calculated in absolute and relative frequencies.

## RESULTS

We evaluated 24 shoulders and 17 hips, in a total of 16 patients with a clinical diagnosis of PMR (10 patients underwent MRI of both shoulders, and seven underwent MRI of both hips). The imaging features are summarized in Table 4.

**Table 4:** MRI fi ndings in patients with PMR.

Imaging findings	n (%)	Bilateral n (%)
Shoulder (n = 24)*		
SASD bursitis	21 (87.5)	8 (80)
Joint effusion	17 (70.8)	8 (80)
Fluid-distended LHB tendon sheath	15 (62.5)	6 (60)
Peritendinitis	21 (87.5)	9 (90)
Capsular edema	17 (70.8)	7 (70)
Hip (n = 17)†		
Joint effusion	12 (70.6)	5 (71)
Trochanteric bursitis	11 (64.7)	2 (28)
Peritendinitis	17 (100)	7 (100)
Capsular edema	14 (82.4)	5 (71)
Spine MRI (n = 6)		
Lumbar interspinous bursitis	4 (66.7)	—
Cervical interspinous bursitis	2 (33.3)	—

* Only 10 of these patients underwent bilateral examination.

† Only 7 of these patients underwent bilateral examination.

**Figure 1:**
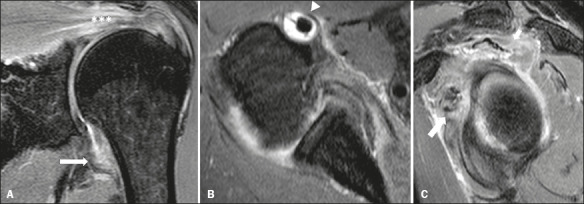
MRI of the left shoulder in a 70-year-old male with PMR. **A**: Coronal T2wFS image showing mild joint effusion with marked capsular and pericapsular soft-tissue edema (arrow). Note also the supraspinatus peritendinitis (asterisks). **B**: Axial T2wFS image showing effusion around the LHB tendon (arrowhead). **C**: Sagittal T2wFS image showing peritendinitis involving the supraspinatus muscle (short arrow) and subscapularis muscle (long arrow).

**Figure 2:**
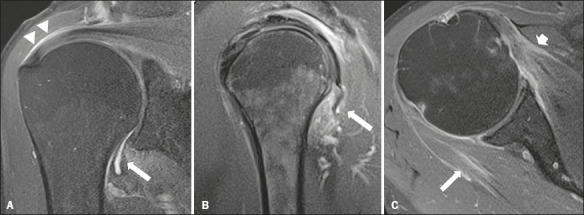
MRI of the right shoulder in a 60-year-old female with PMR. **A**: Coronal T2wFS image showing joint effusion (arrow) and a small amount of fl uid distending the SASD bursa (arrowheads). **B**: Sagittal T2wFS image showing peritendinitis involving the teres minor tendon and muscle (arrow). **C**: Axial T2wFS image showing peritendinitis involving the infraspinatus muscle (long arrow) and subscapularis muscle (short arrow).

As illustrated in Figures 1 and 2, we identifi ed SASD bursitis in 21 (87.5%) of the 24 shoulders evaluated, as well as glenohumeral joint effusion in 17 (70.8%), fl uid distention of the LHB tendon sheath in 15 (62.5%), peritendinitis in 21 (87.5%), and capsular edema in 17 (70.8%). Of the 10 patients who underwent MRI of both shoulders, eight (80%) were found to have bilateral SASD bursitis, eight (80%) were found to have bilateral glenohumeral joint effusion, six (60%) were found to have bilateral fl uid distention of the LHB tendon sheath, nine (90%) were found to have bilateral peritendinitis, and seven (70%) were found to have bilateral capsular edema.

**Figure 3:**
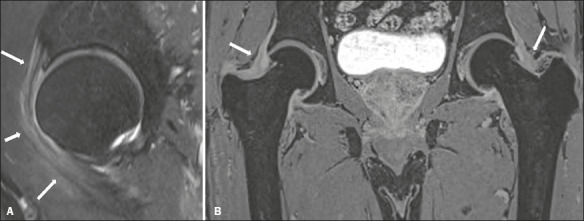
MRI of the right hip in a 58-year-old female with PMR. **A**: Sagittal T2wFS image showing capsular and pericapsular soft-tissue edema (arrows). **B**: Contrast-enhanced coronal T1-weighted fat-saturated sequence showing diffuse enhancement of the joint capsule (arrows).

**Figure 4:**
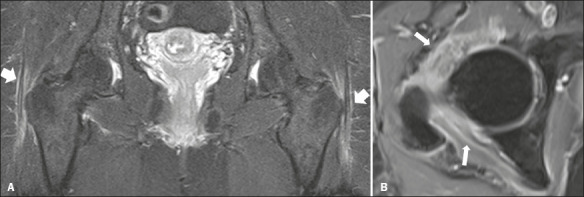
MRI of the hips in a 72-year-old female with PMR. **A**: Coronal T2wFS image showing peritendinitis involving the fascia lata tendon (arrows). **B**: Axial T2wFS image showing capsular and pericapsular soft-tissue edema (arrows).

As illustrated in Figures 3 and 4, hip joint effusion was observed in 12 (70.6%) of the 17 hips evaluated, whereas trochanteric bursitis was observed in 11 (64.7%), capsular edema was observed in 14 (82.4%), and hip peritendinitis was observed in all 17 (100%). Of the seven patients who underwent MRI of both hips, fi ve (71%) were found to have bilateral hip joint effusion, two (28%) were found to have trochanteric bursitis, fi ve (71%) were found to have bilateral hip capsular edema, and all seven (100%) were found to have bilateral hip peritendinitis.

Contrast-enhanced images were acquired in only three patients and did not alter the results of the evaluation of the periarticular soft tissue, because, in all patients, radiologists were able to detect peritendinous and capsular edema on T2wFS images. However, there was marked peritendinous and capsular enhancement even in the patients who presented mild periarticular and capsular edema (Figure 3), which increased the certainty of this fi nding.

**Figure 5:**
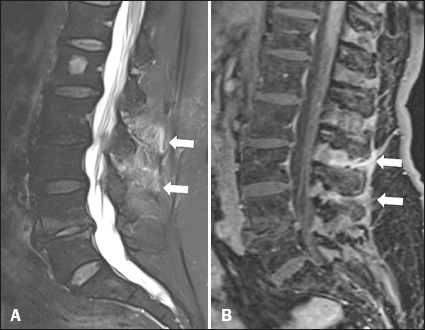
MRI of the lumbar spine in a 70-year-old patient with PMR. **A**: Sagittal T2wFS image showing edema and bursitis of the interspinous ligaments (arrows). **B**: Contrast-enhanced sagittal T1-weighted fat-saturated sequence showing marked enhancement of the interspinous ligaments (arrows).

Of the 16 patients evaluated, six (37.5%) underwent MRI of the spine, four undergoing MRI of the lumbar spine and two undergoing MRI of the cervical spine. All six of those patients were found to have interspinous bursitis (Figure 5).

After the imaging examinations, the patients were treated with oral prednisone. All of them responded to the treatment and showed a reduction in the levels of infl ammatory markers, as well as substantial clinical improvement.

## DISCUSSION

Shoulder abnormalities, especially SASD bursitis and glenohumeral synovitis, are the most common features described in patients with PMR^(7,11,13)^ and are often bilateral**( 14).** In the present serie of cases, nearly 90% of the patients had SASD bursitis. Salvarani et al.^(15)^ found that to be present in 100% of patients with PMR, compared with 22% of control patients. Ochi et al.^(10)^ reported that the degree of fl uid accumulation in the shoulder bursa was signifi cantly higher in patients with PMR than in those with RA. Even in patients with a normal ESR, SASD bursitis represents a hallmark of PMR^(8)^. In a study employing ultrasound and MRI of the shoulders, Cantini et al.^(13,16)^ found SASD bursitis to be the most common fi nding in patients with PMR.

We observed glenohumeral joint effusion in over 70% of the shoulders evaluated, which is in agreement with the fi ndings of studies showing a high prevalence of glenohumeral joint effusion and shoulder synovitis in patients with PMR^(7,11,14)^. However, some authors have shown that the frequency of glenohumeral joint effusion does not differ signifi cantly between patients with PMR and healthy controls**( 8,16)** or between patients with PMR and those with other rheumatic diseases^(9,15)^, suggesting that this fi nding is highly nonspecifi c.

Biceps tenosynovitis is also a classical imaging feature of PMR^(10)^ and has been shown to be present in 62% of shoulders. However, Cantini et al.^(13)^ showed that it is also a common fi nding in patients with other rheumatic illnesses, such as RA and psoriatic arthritis. In another study, Cantini et al.^(16)^ reported that there was no difference between patients with PMR and controls in terms of the prevalence of biceps tenosynovitis.

Only a few studies have evaluated hip abnormalities in PMR^(10,17)^. Cantini et al.^(17)^ analyzed hip MRI in patients with PMR and identifi ed hip joint effusion in up to 85%. That is in accordance with the fi ndings of the present study, in which 70% of the hips showed some degree of joint effusion. However, Ochi et al.^(10)^ found no signifi cant difference between patients with PMR and those with RA in terms of the degree of hip joint effusion.

Trochanteric bursitis is another feature commonly described in PMR. Cantini et al.^(17)^ found that 100% of patients with PMR had trochanteric bursitis, compared with 64,7% of our patients. Ochi et al.^(10)^ also showed that the amount of effusion in hip bursae was greater in patients with PMR than in patients with RA and in controls.

Although prevalent, bursitis and synovitis of the shoulder or hip are not specifi c for PMR. They are commonly found in the imaging examinations of patients with RA^(9,11,16)^ and even in those of healthy elderly patients^(8)^, in whom they are mostly related to mechanical or degenerative changes.

Another consistent fi nding in our study was capsular edema, which was seen in over 70% and 80% of the shoulders and hips evaluated, respectively. That is in contrast with the fi ndings of some studies that suggest that synovitis of the bursae and joints is the only factor responsible for the initial localization of PMR^(15,18,19)^. However, most of those studies used ultrasound, which is less sensitive to extracapsular changes. The authors of studies conducted more recently and using MRI have reported fi ndings similar to ours and have stated that periarticular soft-tissue edema is a characteristic fi nding of PMR^(7,10,11,20)^, suggesting that it is responsible for the severe discomfort and myalgia that radiate toward the periphery in these patients.

Ochi et al.^(10)^ showed that periarticular soft-tissue edema, in shoulders and hips, was more common among patients with PMR than among patients with RA, stating that it can therefore facilitate the differential diagnosis between those two rheumatic conditions^(11)^. Another study evaluating patients with PMR and patients with RA also showed that all of the patients with PMR had extracapsular enhancement in their hand joints, compared with only half of those with RA^(9)^. Those fi ndings suggest that infl ammation is more pronounced in periarticular tissues than in the synovia and that the anatomical basis of the initial location of the disease differs between RA and PMR.

One major differential diagnosis of pericapsular softtissue edema of the shoulder is adhesive capsulitis, a wellknown condition that involves the axillary recess and the rotator interval of the shoulder^(21)^. The differentiation between PMR and adhesive capsulitis relies not only on clinical and biochemical data but also on imaging fi ndings. Periarticular infl ammatory involvement is usually more extensive in PMR, involving the tendon and muscle bellies, than in adhesive capsulitis^(7)^. However, if the relevant clinical data are unavailable, this differential diagnosis can be challenging.

Interspinous bursitis has been implicated as the cause of cervical and lumbar discomfort in patients with PMR^(22,23,24)^. All six of the patients who underwent MRI of the spine in our study showed some degree of interspinous bursitis, in the lumbar or cervical spine.

## CONCLUSION

Imaging features such as SASD bursitis, glenohumeral joint effusion, and hip joint effusion are highly prevalent in patients with PMR, as are peritendinitis and capsular edema. There is a need for case-control studies to confi rm our findings and to determine the real impact that these findings have on the diagnosis of PMR by MRI.
